# Supplementary material for: Trial-level sequence modeling reveals hidden dynamics of dual-task interference
Source: PLoS Comput Biol. 2026 May 20;22(5):e1014302. doi: 10.1371/journal.pcbi.1014302 (PMC13215611; doi:10.1371/journal.pcbi.1014302)
Supplement: S1 Appendix — Contains analysis of the clusters found in the projection of response operation embeddings. (PDF) [file pcbi.1014302.s001.pdf]

1 Response operation cluster analysis

While analyzing the embeddings used in Fig 3 of the main text, we found that across dimensionality reduction methods (T-SNE, PCA, UMAP), the response operation was consistently clustered into sub-clusters. This indicates that there are sub-groupings within the *Response* operation that are representationally distinct. That the model embeds these into different parts of embedding space indicates that it benefits from this grouping when classifying. Initially, we expected to find an experimental factor that explained these clusters, such as response modality (which finger was used by the participant), or which stimulus was presented (combination of flanker + central color). Alternatively, a behavioral measure that the model has either implicit or explicit access to could explain the clusters, such as reaction time (RT), epoch index, or the trial's position within the block. Finally, some methodological or model bias could exist that caused the clusters. To examine the cause of the clusters, we first used the clustering algorithm HDBSCAN [1] ($min\_cluster\_size = 53$, chosen based on visual separation of clusters) to separate the projected embeddings (Fig 1a) into defined clusters (Fig 1b). By training a Random Forest classifier to predict the clusters based on different factors, we attempted to find the variable that best explained the clusters. Analysis showed that the difference in samples between the predicted peak time of the *Response* operation and the reaction time best explained the cluster structure (Fig 1c), with a five-fold cross-validated Random Forest classifier achieving a macro F1-score of 0.984 (baseline macro F1-score on shuffled cluster labels: 0.051; chance level 12.5%). We interpret these findings as a learned binning of the duration of the *Response* operation, as we see the peak predicted time as the onset of the *Response* operation, and the RT is essentially the end of this operation. Intuitively, if a greater difference between *Response* onset and RT indicates a longer *Response* operation, there is more *Response*-related activity in the averaged 13 embeddings, which we believe is what the model is picking up. If, however, the *Response* duration was static, there is no need for the model to cluster the embeddings, as the amount of non-*Response* activity is constant. Interestingly, we observe that the amount of clusters decreases with a smaller embedding window, which is explained by the fewer possible sample difference amounts (and thus *Response*-related activity bins). Additionally, we expect that this effect is dependent on the sampling rate. If we increased the sampling rate (and window size proportionally), we would expect it to be more difficult for the model to determine sample-to-sample whether the *Response* operation has ended, since sample-to-sample differences are then smaller. One might wonder why these sub-clusters occur in the *Response* operation and not in the *Encoding* or *Central* operations, since these also end at different points. Our interpretation is multifaceted, firstly, since the *Response* operation is at the end of a trial, task-related activity is increasing continuously and is suddenly not needed anymore, possibly resulting in a detectable change in electroencephalogram (EEG). Secondly, gradients in a deep network accumulate towards the end of a trial, meaning that it might be helpful for the model to learn features that dissociate classes at the end of a trial more clearly.

References

1. McInnes L, Healy J, Astels S. Hdbscan: Hierarchical Density Based Clustering. Journal of Open Source Software. 2017 Mar;2(11):205. doi:10.21105/joss.00205.

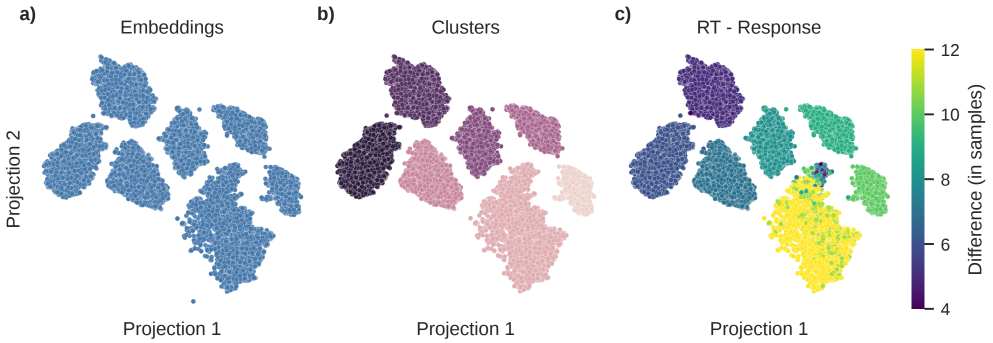

Fig 1. Cluster analysis. a) 2-D T-SNE projection of Task 1 *Response* operation embeddings. **b)** HDBSCAN-clustered cluster labels, with small, noisy clusters left out. **c)** Difference between RT and *Response* operation prediction in samples, color bar min and max chosen based on means of clusters.
