# Supplementary material for: Trial-level sequence modeling reveals hidden dynamics of dual-task interference
Source: PLoS Comput Biol. 2026 May 20;22(5):e1014302. doi: 10.1371/journal.pcbi.1014302 (PMC13215611; doi:10.1371/journal.pcbi.1014302)
Supplement: Attachment — Submitted filename: Response to reviewers.pdf [file pcbi.1014302.s003.pdf]

We thank the editors and reviewers for their careful reading of our manuscript and their well thought-out feedback. We have revised our manuscript accordingly and provide below a point-by-point response. All changes are highlighted in the revised manuscript, as requested. We hope the revised manuscript addresses the reviewers' concerns.

Reviewer 1

> The paper uses EEG data to analyze two issues of relevance to studies of the psychological refractory period (PRP). The data involve a short SOA condition where Task 2 begins just 300 msec after Task 1 and Task 1 typically has not been completed and a long SOA condition where Task 2 begins 1000 msec after Task 1 and Task 1 typically has been completed. The two questions are (1) whether the stages of the two tasks remain the same in the two conditions and (2) whether it is really true that performance of Task 1 is not changed in the short SOA condition.

> The answer to the first question is not compelling, as detailed below. The answer to the second question has a potential confusion of causal direction, also as detailed below.

Comment 1

> The paper uses hidden multivariate pattern (HMP) analysis to identify the stages in the task. The analysis focuses on the EEG data from the onset of the task to 50 msec after the task response and identifies 50 msec event patterns in the data that mark stages in processing. Four analyses are performed – of both tasks at both SOAs. The paper reports three event patterns in all 4 analysis which are labeled as Encoding, Central, and Response. A more thorough reporting of the 4 analyses is needed. We are only shown the Figure 2 summary of the two tasks in the long condition. There is no evidence that there are these three event patterns, not more or less. Such confirmation of number of event patterns is common in HMP analysis.

Reply: The reviewer raises two important questions. Firstly, we recognize the need to clarify how our HMP analysis was performed. As the reviewer states, it is indeed important to report on all HMP fits that were performed. In our study, we performed only two HMP analyses, one for Task 1 in the Long SOA condition, and one for Task 2 in the Long SOA condition. We did not perform HMP analyses on the Short SOA condition, since HMP fails to provide a good fit when the temporal dependence of subsequent events is disturbed, as is the case in our Short SOA condition. We see that this requires better clarification in the Methods section, which we have now added (2.3 Hidden Multivariate Pattern Analysis).

Updated text:

> *In the current application, we fit HMP to the Long condition only. This is necessary because HMP relies on the temporal dependence of subsequent events [35]. In the Short SOA condition, this temporal dependence may not be present due to Task 2 operations occurring while Task 1 is being performed. With a Long SOA of 1200 ms, Task 1 and 2 overlap is negligible, as the 95th percentile of Task 1 RT is at 840 ms, which is substantially shorter than the Long SOA . Application of HMP to the Long SOA condition allows us to estimate the onset of the cognitive operations of each task reliably and independently*

Reply: Secondly, regarding the reviewer's point on confirming the number of event patterns, we used HMP's Cumulative fitting method which leaves the number of events as a free parameter to be optimized. This means that the number of events is determined by the fitting procedure and the event width parameter (determining the event pattern size). For this parameter, we tested a range of values and chose the value that provided the most stable solution, as determined by it and its neighboring values resulting in the same number of events. We added more information regarding this process to the manuscript in section 2.3: Hidden Multivariate Pattern Analysis, including the limits of numbers of events that were estimated at different event widths.

Updated text:

> *We tuned the event width parameter by testing a range of different event widths (20-60 ms, steps of 5 ms, cf. [35]). The event width fits ranged from six Task 1 and six Task 2 events (when the event width was 20 ms), to three Task 1 and two Task 2 events (when the event width was 60 ms). An event width of 50 ms – giving three events – was determined as the most stable value by investigating the number of estimated events, meaning that this value and the surrounding event widths (45 ms and 55 ms) all resulted in three events in both tasks.*

Comment 2

> The first question is whether the event patterns are the same for the two SOAs. One might have expected a standard statistical comparison of electrode values or PCA values associated with the event patterns. However, a deep learning architecture is used to extract embeddings associated with the event patterns and examine the similarity between long and short embeddings. It is not clear (to me) in detail what the embeddings mean. Figure 3 does not really help. What does "Other" in part B refer to? The caption refers to "other operations" – are these other operations in the same task or the other task. To the extent that there is a statistical conclusion it is that same distances are smaller than "other" distances. However, one would want to know whether the differences between the same operations in short and long is statistically different from 0.

Reply: The reviewer raises an essential question regarding the interpretation of embeddings. Conceptually, PCA components and embeddings serve related roles. PCA provides a linear decomposition of the data, primarily reflecting spatial covariance structure in the EEG. In contrast, the embeddings as used here reflect the sequence model's learned transformation of the EEG into a shared latent representational space that integrates spatial, temporal, and potentially higher-order features. Therefore, embeddings provide a rich, data-driven representation of cognitive operations.

Although embeddings are less directly interpretable than PCA components, they provide a data-driven representational similarity metric. This is particularly important in the short SOA condition, where (as mentioned earlier) overlapping task activity makes it difficult to isolate cognitive operations using just HMP. Because our approach requires using the sequence model to identify operations in the Short condition, embeddings provide an internally consistent way to probe operation representation similarity across conditions.

We apologize for the lack of clarity regarding our "Same" and "Other" values. "Other" refers to other cognitive operations within the same task, not operations from the other task. This is reflected in the separate 'Task 1' and 'Task 2' columns in Figure 3. We have clarified this in the caption of Figure 3.

Updated text:

> *the absolute cosine distance difference (Y-axis) between embeddings of cognitive operations in the Short condition and those same operations in the same task (X-axis) in the Long condition is negligible (95%CI includes 0), and smaller than the difference between operations in the Short condition, and the other operations in the same task (X-axis) in the Long condition.*

Reply: The reviewer also asks whether same-operation Short vs Long differences are statistically different from zero. This is in fact what our analysis shows. The distribution of same-operation cosine distance differences is near-zero, with median = 0.00 and 95% CI [0.00, 0.02], indicating no meaningful representational difference between conditions. We have clarified our interpretation in the revised manuscript in section 3.2: Cross-decoding cognitive operations across condition.

Updated text:

> *The cosine distance to other operations, within task, was larger (median = 0.50, 95% CI [0.26, 0.54]), as demonstrated by the non-overlapping confidence intervals. These results show*

> *separation in cosine similarity between sets, indicating no meaningful difference between operations of the same task across delay conditions.*

Comment 3

> The second question is whether the first task is unchanged in the short condition. To do this separate HPM analyses are performed of the overlapping EEG periods for the two tasks. In the short SOA condition, one might have expected an analysis of the single period from the beginning of the first task to the end of the second task, looking for event patterns from the two tasks. The paper's approach of separate HPM analyses offers some advantages, but one has to worry about a event pattern from the one task being attributed to the other.

Reply: We thank the reviewer for raising this important point. We agree that when the two tasks overlap in time, there is a potential concern that event patterns associated with one task could be misattributed to the other by our sequence model.

HMP can only be applied successfully to situations where both tasks are separate in time, such as in our Long SOA condition. In this step, there is no possibility of the HMP model confusing event patterns between tasks, since the tasks do not overlap. This is supported by the observation that the 95th percentile of the RT distribution of Task 1 is 840 ms, which is well before the 1200 ms SOA in the Long SOA condition. We have added this information to the manuscript in Section 2.3: Hidden Multivariate Pattern Analysis.

Updated text:

> *With the Long SOA of 1200 ms, Task 1 and 2 do not overlap (95th percentile of Task 1 RT is 840 ms), which allows us to estimate the onset of the cognitive operations of each task independently.*

Reply: However, in the second part of our methodological pipeline, where we apply a sequence model to the overlapping Short SOA condition, there is a possibility for misattribution. To check whether this was likely we performed additional analyses.

Firstly, note that a misattribution would likely increase the frequency of 'impossible' sequences, for example: E1 > C2 > R1 > E2 > C1 > R1, which is impossible due to the central operation for Task 2 occurring before its stimulus content has started being encoded. In this case the operations are swapped entirely, but of course the same holds for E1 > C2 > C1 > R1 > E2 > R2 and E1 > C1 > C2 > R1 > E2 > R2. Sequences like these, where the inter-task operation order was not valid, occurred 108 times. This accounts for 0.47% of the total trials in the set we used for statistics. We added a sentence in section 3.3: "Decoding the sequence of cognitive operations across tasks" to reflect this.

Updated text:

> *Here, we excluded invalid sequences (e.g., R1 → E1 → C1 → C2 → E2 → R2), which occurred only marginally (0.47% of trials, see Table 1 for sequence definitions)*

Reply: Secondly, in sequences such as those mentioned earlier, when the model misattributes an operation belonging to one task to the other, one would expect the temporal distance between those operations to be near zero, because the model for Task 1 would look for the same pattern as the model for Task 2, and thus provide a similar prediction timing. We analyzed these same-operation cross-task differences (including ALL predicted sequences) and found that they were mainly distributed above 0, see Figure R1. This means that for Encoding, Central, as well as Response, the attributed order of the tasks was consistent and aligned with our hypothesis.

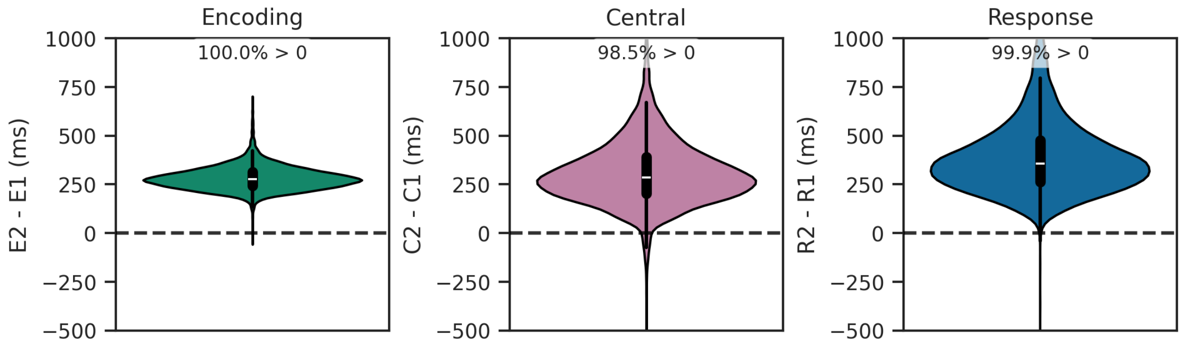

Figure R1

Comment 4

> The HMP gives the most probable location of the event pattern on each trial, and I assume this is what produces the sequences for Table 1 and Figure 4. The sequences are the orders of those most probable locations. Given the nature of the HPM and the fact that task 1 finishes before task 2 starts in the long condition there is only one possible ordering of conditions: E1 → C1 → R1 → E2 → C2 → R2. However, there are 6 sequences in the short condition counting the ignored: E1 → E2 → C2 → C1 → R1 → R2. The duration and accuracy of Task 1 varies with the sequence. The different sequence are interpreted as different strategies employed by the participant. However, the sequences can be viewed as just where the stages of one tasks fell compared to the independent stages of the second task. There is something of a speed-accuracy trade-off in Task 1. The only cases where one could get the E1 → C1 → R1 → E2 → C2 → R2 sequence in the Short SOA is when task 1 completes near the beginning of when task 2 is presented (300 msec. later). It is a necessity that this sequence is associated with brief response time and responding so rapidly seems the reason for the low accuracy.

Reply: The reviewer poses a valid concern regarding the potential direction of causality between sequence structure and behavior. We agree with the reviewer that there is a general speed-accuracy trade-off in Task 1, and that the sequence E1 → C1 → R1 → E2 → C2 → R2 must logically result in shorter reaction times. This is because this sequence can only occur when Task 1 processing is largely completed before Task 2 overlap occurs.

However, we interpret the data as showing that sequence differences reflect more than independent stage timing or overall Task 1 response speed. In the PRP paradigm, task processing is inherently interdependent, as reflected in the classical PRP effect, where Task 2 processing is delayed by Task 1. Our results extend this by showing that Task 1 behavior is also modulated by interference structure, which is typically not observed because behavioral analyses average across heterogeneous trial-level dynamics.

Importantly, if sequences only reflected a speed-accuracy trade-off, one would expect a monotonic relationship between Task 1 RT and accuracy across sequences. Instead, we observe that this trade-off is not consistent across sequences (e.g., comparisons B vs C, C vs D, and D vs E), where longer RTs do not necessarily show reduced accuracy. This suggests that, beyond general speed-accuracy trade-off effects, the relative timing and ordering of cognitive operations across tasks differentially affects behavior.

We therefore interpret sequences as reflecting trial-level variations in how processing of the two tasks interacts over time, rather than purely independent timing of fixed stages of processing. We use the term "strategy" descriptively, denoting repeatable patterns of temporal organization, without implying conscious or deliberate control. Lastly, we do not claim that sequences are independent of RT, but rather that they capture temporal structure beyond what is explained by RT alone. We added sentences to Section 4: Discussion to reflect these points.

Updated text:

> *Here, a strategy refers to repeatable patterns of temporal organization, without implying conscious or deliberate control. In our analysis, this repeatable pattern is a sequence of cognitive operations. However, we find an effect on Task 1 RT that depends on the specific strategy used. We interpret this as evidence that the sequence structure we identify captures aspects of temporal structure that are not fully reflected in RT alone.*

Comment 5

> It is a mistake to treat these sequences as causing behavioral differences and ignore the likely possibility that it is differences in the speed of performance of the two tasks that are producing the different sequences. Also, as the authors admit, it is deeply problematic to take the maximum likelihood locations as reflecting when the operations are performed. The locations are far from certain and presumably there are many cases where the order of two event patterns could have been flipped without much cost to likelihood.

Reply: We agree with the reviewer that taking the maximum likelihood (peak) locations as the single time-point at which the operation occurs underestimates uncertainty, as it does not account for the uncertainty in our method's estimation of the cognitive operations. To address this, we extended our analysis with a probabilistic sampling approach. Here, we sample event onset times from the full predicted distribution, weighted by the probabilities. Instead of assigning each epoch to a single sequence, we now derive a posterior distribution over all plausible sequences, while enforcing within-task ordering constraints to exclude cognitively implausible orders. Note that due to the new probabilistic sampling approach giving better behavioral estimates for each sequence, we are also able to analyze sequence F, previously excluded due to limited statistical power.

Due to the newly introduced uncertainty in sequence assignments, we also adjusted our behavioral analyses. Specifically, we repeatedly sampled complete datasets from the epoch sequence posteriors, refit the RT and accuracy mixed-effects models for each sampled dataset, and combined parameter estimates using Rubin's rules for multiple imputation. This procedure directly tests whether our behavioral conclusions depend on point estimates of event timing. The results can be seen in Figure 4 of the revised manuscript (and in Figures R2 and R3 below for ease of access).

Although the estimated proportions of sequences change under probabilistic assignment, the key behavioral patterns and statistical conclusions remain similar. We have updated the Methods and Results to describe this procedure and report the behavioral analyses. See Figures R2 (old) and R3 (new) for comparison of results

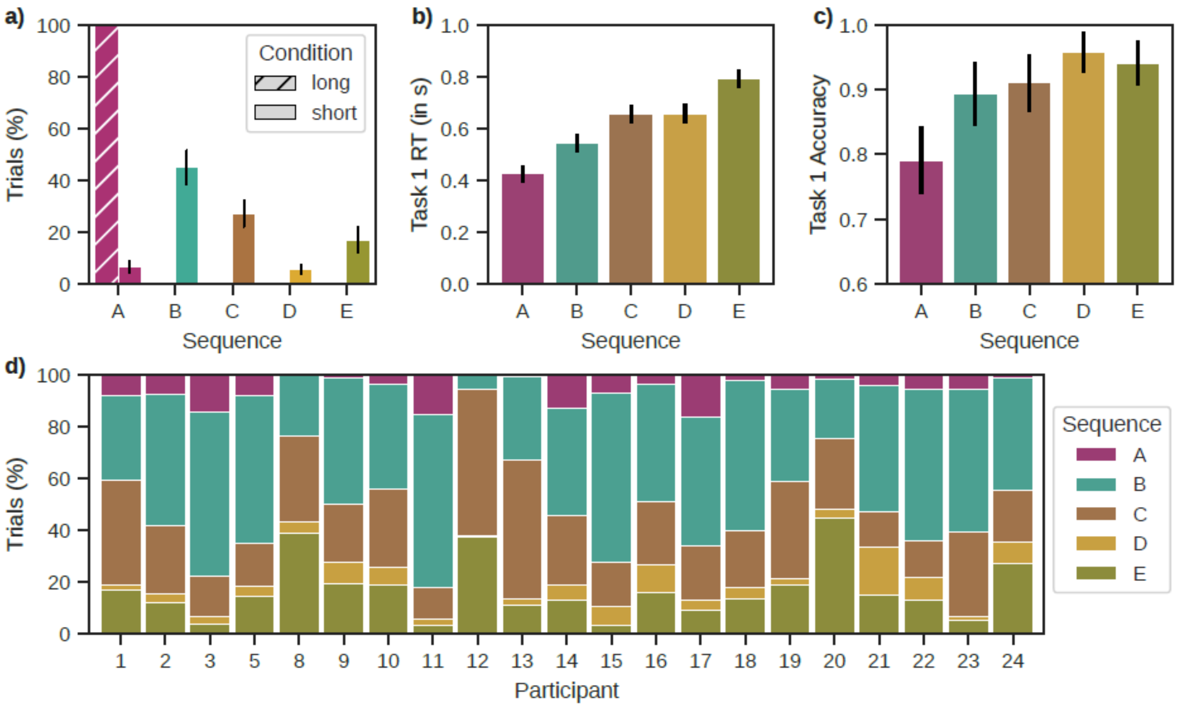

Figure R2

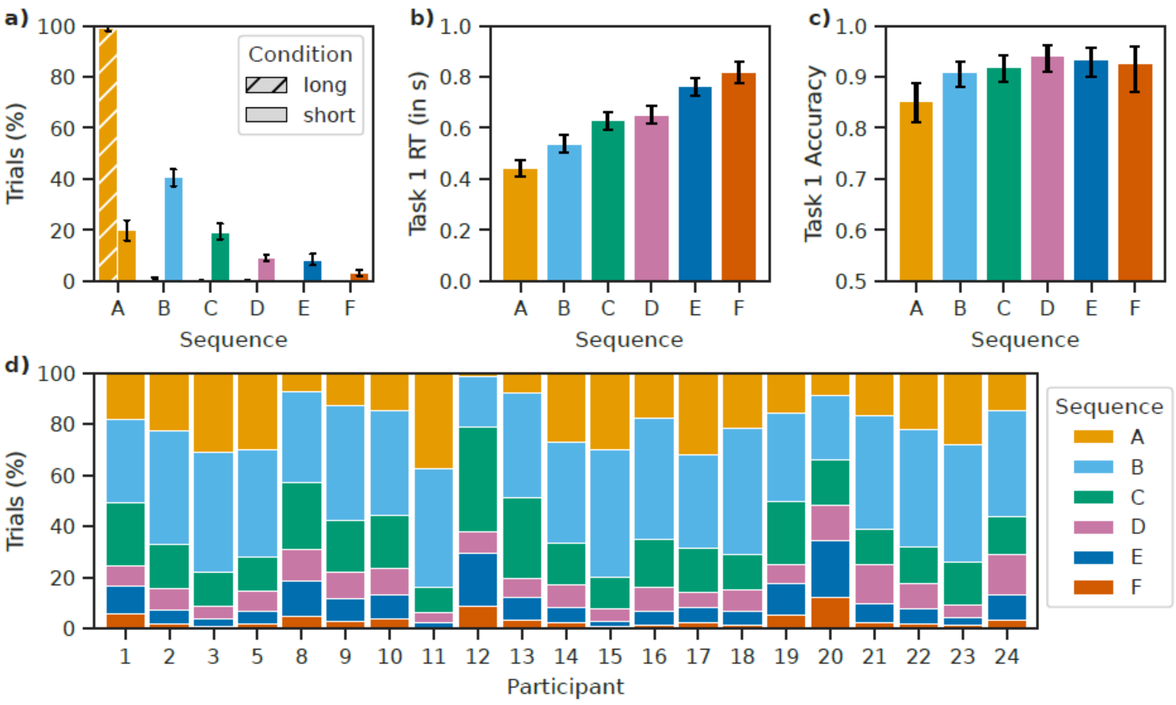

Figure R3

Reviewer 2

> The present study investigated the processing architecture and processing strategy of task performance in the PRP paradigm. While previous studies applied RT data, imaging data, EEG data, etc. in an average way, the present study investigated PRP task performance on a trial level. In particular, the processing stages of encoding, central, and response execution were separated, and the processing sequence of these stages was analysed in a re-analysis of an existing data set of Steinhauser and Steinhauser. The authors found individual trial-by-trial variability related to strategies that directly affect accuracy and RTs. The study discusses these findings in the context of tradition and more modern theories on multitasking.

> I like the methodological approach of the manuscript and the conclusions that can be derived from the HMP method. However, before recommending this manuscript for publication, there are some

> substantial comments to solve. These comments are rather from a theoretical than from a methodological perspective and listed in the order of the structure of the manuscript.

Comment 1

> Line 20: “In contrast, Task 2 responses are typically delayed.” This sentence is not correct in itself in the context of PRP dual tasks. It is the long versus the short Task 2 condition which is delayed, and which is correctly stated in the following sentence. Therefore, I suggest cutting this sentence (“In contrast, Task 2 ...”).

Reply: We thank the reviewer for pointing out this theoretical inconsistency and have adjusted the manuscript by removing the sentence ("In contrast, Task 2...")

Comment 2

> Lines 46 – 48: “For example, the PRP effect can decrease with practice [18, 19], possibly indicating a shift from serial to parallel processing [20], although it can be argued that people simply become fast at each task separately and thus encounter costs of parallel execution less often [21].” Please, refer to more recent research on practice effects such as Strobach & Schubert (2017: Strobach, T., & Schubert, T. (2017). No evidence for task automatization after dual-task training in younger and older adults. Psychology and Aging, 32(1), 28-41.) or Schubert et al. (in press: Schubert, T., Liepelt, R., & Strobach, T. (in press). Evidence for a latent bottleneck after extensive dual-task practice of a visual-manual and an auditory-verbal task. Quarterly Journal of Experimental Psychology.)

Reply: The reviewer rightfully points out more recent research reporting smaller practice effects than implied in the manuscript. We have adjusted the relevant sentence to state that the PRP effect does not disappear under practice and included the cited references in the updated manuscript.

Updated text:

> *For example, the PRP effect can decrease with practice [18, 19] (but does not disappear [20, 21])*

Comment 3

> Line 68: At this point, it is relevant to review relevant EEG literature on PRP dual tasks. How is previous EEG research investigating PRP dual tasks? What are the investigated task components? What are the main findings of this field? What are the limitations? Please, also refer to Töllner et al. (2012: Töllner, T., Strobach, T., Schubert, T., & Müller, H. (2012). The effect of task order predictability in audio-visual dual task performance: Just a central capacity limitation? Frontiers in Integrative Neuroscience, 6:75.)

Reply: We agree with the reviewer that specific EEG literature on PRP dual-tasks is missing from the introduction, and have added text to the introduction on which EEG/ERP components have traditionally been used to investigate dual-task performance.

Updated text:

> *A body of electroencephalogram (EEG)/event-related potential (ERP) research has examined the neural dynamics of PRP dual-task performance. A body of electroencephalogram (EEG)/event-related potential (ERP) research has examined the neural dynamics of PRP dual-task performance. These studies typically track task-specific components associated with perceptual processing (N2pc, posterior contralateral negativity (PCN)) and response preparation (lateralized readiness potential (LRP)). Consistent with the central bottleneck theory, several studies report that central processing affects ERP components related to perceptual [31–33] and motor [33] processing. A potential limitation of this work is that it relies on trial-averaged ERPs and thus assumes a fixed temporal structure across trials.*

Comment 4

> In general, I think that the manuscript would strongly benefit by applying the methodological approach of HMP to another data set. So far, the conclusions are exclusively based on a single experimental set of data from 24 participants. This manuscript would benefit a lot, when the current findings could be replicated and specified, and in this way, the manuscript could make a strong impact in the field.

Reply: We consider the reviewer's comment a valuable remark, demonstrating generalization across datasets would further strengthen the manuscript. Unfortunately, we did not succeed in finding a suitable dataset. Specifically, to assess suitability of datasets, we explored suitable open EEG datasets in the PRP paradigm. Because our approach requires a sufficiently long SOA to obtain trials in which Task 1 processing is completed before Task 2 onset, the number of publicly available datasets meeting these constraints is limited. We examined datasets from the following papers:

1. Töllner, T., Strobach, T., Schubert, T., & Mueller, H. J. (2012). The effect of task order predictability in audio-visual dual task performance: Just a central capacity limitation? *Frontiers in Integrative Neuroscience*, *6*. https://doi.org/10.3389/fnint.2012.00075
2. Rabovsky, M., Conrad, M., Álvarez, C. J., Paschke-Goldt, J., & Sommer, W. (2019). Attentional modulation of orthographic neighborhood effects during reading: Evidence from event-related brain potentials in a psychological refractory period paradigm. *PLOS ONE*, *14*(1), e0199084. https://doi.org/10.1371/journal.pone.0199084
3. Corriveau, I., Fortier-Gauthier, U., Pomerleau, V. J., McDonald, J., Dell'Acqua, R., & Jolicoeur, P. (2012). Electrophysiological evidence of multitasking impairment of attentional deployment reflects target-specific processing, not distractor inhibition. *International Journal of Psychophysiology*, *86*(2), 152–159. https://doi.org/10.1016/j.ijpsycho.2012.06.005
4. Roberge, A., Duncan, J., Fiset, D., & Brisson, B. (2019). Dual-Task Interference on Early and Late Stages of Facial Emotion Detection Is Revealed by Human Electrophysiology. *Frontiers in Human Neuroscience*, *13*. https://doi.org/10.3389/fnhum.2019.00391
5. Li, Q., Wang, J., Meng, Z., Chen, Y., Zhang, M., Hu, N., Chen, X., & Chen, A. (2024). Decoding the task specificity of post-error adjustments: Features and determinants. *NeuroImage*, *297*, 120692. https://doi.org/10.1016/j.neuroimage.2024.120692

For papers 1 (150/300/600 ms), 2 (700ms), and 3 (150/450/900 ms), the SOA values were short to the point where RT to Task 1 was on average always higher than the SOA, which would result in too many exclusions. For reference, our mean Task 1 RT is 571 ms in the Long SOA condition, which ends after 1200 ms. Paper 4 would be a better match, as they include a 1000 ms SOA condition where the mean RT to Task 1 is only marginally higher. The long SOA of paper 5 is 2000 ms, which would work perfectly. We contacted the authors of the last two listed studies, but unfortunately did not receive access to the underlying data.

While a full cross-dataset replication proved infeasible in the context of the current manuscript, we have taken several steps to strengthen the robustness of our within-dataset claims. In particular, following Reviewer 1's suggestion, we replaced peak-based sequence assignment with a probabilistic sampling framework and showed that the key behavioral results remain stable when uncertainty in event timing is explicitly kept throughout the analysis.

We have added a discussion of the SOA limitations and the future work of cross-dataset validation to our discussion.

Updated text:

> *Lastly, our methodological approach requires the Long condition to have a sufficiently large SOA value, combined with a sufficiently quick Task 1, to ensure that there is no task overlap on sufficient trials. However, experiments deploying the PRP paradigm often do not include a long*

> *enough SOA [32, 33, 61] to ensure the absence of task overlap. This limits the cross-dataset generalizability of our approach.*
